# Late-onset opportunistic infections while receiving anti-retroviral therapy in Latin America: burden and risk factors

**DOI:** 10.1016/j.ijid.2022.06.041

**Published:** 2022-06-26

**Authors:** Isaac Núñez, Brenda Crabtree-Ramirez, Bryan E. Shepherd, Timothy R. Sterling, Pedro Cahn, Valdiléa G. Veloso, Claudia P Cortes, Denis Padgett, Eduardo Gotuzzo, Juan Sierra-Madero, Catherine C. McGowan, Anna K. Person, Yanink Caro-Vega

**Affiliations:** 1 Instituto Nacional de Ciencias Médicas y Nutrición Salvador Zubirán, Departamento de Infectología, Ciudad de México, México; 2 Vanderbilt University Medical Center, Nashville, TN, USA; 3 Fundación Huésped, Buenos Aires, Argentina; 4 Instituto Nacional de Infectología Evandro Chagas, Rio de Janeiro, Brazil; 5 Universidad de Chile-Fundación Arriarán, Santiago, Chile; 6 Hospital Escuela Universitario, Tegucigalpa, Honduras; 7 Instituto de Medicina Tropical “Alexander von Humboldt,” Universidad Peruana Cayetano Heredia, Lima, Perú

**Keywords:** Opportunistic infections, HIV, AIDS, Latin America, Tuberculosis, Cohort studies

## Abstract

**Objectives:**

The aim of this study was to describe the incidence, clinical characteristics, and risk factors of late-onset opportunistic infections (LOI) in people who live with HIV (PWLHA) within the Caribbean, Central and South America network for HIV epidemiology.

**Methods:**

We performed a retrospective cohort study including treatment-naive PWLHA enrolled at seven sites (Argentina, Brazil, Chile, Peru, Mexico, and two sites in Honduras). Follow-up began at 6 months after treatment started. Outcomes were LOI, loss to follow-up, and death. We used a Cox proportional hazards model and a competing risks model to evaluate risk factors.

**Results:**

A total of 10,583 patients were included. Median follow up was at 5.4 years. LOI occurred in 895 (8.4%) patients. Median time to opportunistic infection was 2.1 years. The most common infections were tuberculosis (39%), esophageal candidiasis (10%), and *Pneumocystis jirovecii* (*P. jirovecii*) pneumonia (10%). Death occurred in 576 (5.4%) patients, and 3021 (28.5%) patients were lost to follow-up.

A protease inhibitor–based regimen (hazard ratio 1.25), AIDS-defining events during the first 6 months of antiretroviral-treatment (hazard ratio 2.12), starting antiretroviral-treatment in earlier years (hazard ratio 1.52 for 2005 vs 2010), and treatment switch (hazard ratio 1.31) were associated with a higher risk of LOI.

**Conclusion:**

LOI occurred in nearly one in 10 patients. People with risk factors could benefit from closer follow-up.

## Introduction

Considerable attention has been focused on late presentation to care of people living with HIV/AIDS (PLWHA) (Bisbal *et al*., 2021; Crabtree-Ramírez *et al*., 2011; Komninakis *et al*., 2018; Mohammadi *et al*., 2021). Virologic failure after antiretroviral therapy (ART) start is more common, retention in care is lower, and mortality is higher for those who present late than for those who present early to care and who thus receive timely ART (Honge *et al*., 2016; Sobrino-vegas *et al*., 2016). These “late presenters” commonly present with an AIDS-defining event (ADE), frequently an opportunistic infection (OI), the relative frequency of which has been previously well described (Crabtree-Ramírez *et al*., 2016). Nonetheless, less attention has been directed to late-onset opportunistic infections (LOIs), especially those that occur after the risk for immune-reconstitution inflammatory syndrome has passed (i.e., usually, the first 6 months after ART start). The incidence of and predictors for these late-onset events have been poorly described.

A few related studies have been performed elsewhere. A Ugandan cohort found that although OIs were most frequent during the first year after diagnosis, almost 30% occurred during the second year or later (Weissberg *et al*., 2018). The most common OI was esophageal candidiasis, followed by pulmonary tuberculosis. In the study, higher viral load was the most prominent risk factor for development of any OI. In an earlier retrospective study performed by our group of outcomes in PLWHA who developed cryptococcal meningitis (CM), the median time to diagnosis of CM was 2 years in the group that developed CM after ART start. Those patients with late-onset CM had higher mortality than the patients who were diagnosed with CM before commencing ART (Crabtree Ramírez *et al*., 2017). Given that higher mortality was found in those later AIDS-related events, we aim to describe the incidence of late-onset AIDS-defining opportunistic infections and associated predictors in PLWHA who are cared for at the Caribbean, Central and South America network for HIV epidemiology (CCASAnet) sites.

## Methods

### Cohort description

The CCASAnet cohort is a part of the International epidemiology Databases to Evaluate AIDS, an international research consortium to address HIV/AIDS research. CCASAnet includes sites in seven Caribbean and Latin American countries and has been described previously (Mcgowan *et al*., 2007). In this study, we included PLWHA aged 18 years and older who were enrolled between 2001 and 2015 at a CCASAnet site: Centro Médico Huésped in Buenos Aires, Argentina (Argentina-CMH); Instituto Nacional de Infectología Evandro Chagas in Río de Janeiro, Brazil (Brazil-INI); Fundación Arriarán in Santiago, Chile (Chile-FA); Instituto Hondureño de Seguridad Social and Hospital Escuela Universitario in Tegucigalpa, Honduras (Honduras-IHSS/HE); Instituto de Medicina Tropical Alexander von Humboldt in Lima, Peru (Peru-IMT); and Instituto Nacional de Ciencias Médicas y Nutrición Salvador Zubirán in Mexico City, Mexico (Mexico-INNSZ). Data from the two sites in Honduras were combined in our analyses. The CCASAnet Haiti site was excluded owing to limited data on clinical outcomes.

### Study definitions and outcomes

Follow-up began at 6 months (i.e., 180 days) after ART initiation; we excluded patients who were lost to follow-up or who died during the first 6 months after ART start, and those whose previous AIDS status was unavailable. Outcomes of interest were AIDS-defining LOIs, loss to follow-up (LTFU), and death. AIDS-defining OIs were classified according to the Centers for Disease Control definition of AIDS (cancers and wasting syndrome were not included), and they were considered late onset if they occurred 180 days or more after starting ART (AIDS and Opportunistic Infections, Centers for Disease Control and Prevention, 2021). LTFU was defined as no visits within 1 year of the database closing date of each center. Follow-up ended with the occurrence of a clinical end point (LOI or death) or the date last seen alive (last clinic visit, registered clinical endpoint, last viral load, or last CD4 count).

Variables for which we evaluated their association with the outcomes were chosen for inclusion *a priori*: age, sex, site, likely mode of HIV transmission, initial ART regimen received (nucleoside reverse transcriptase inhibitors [NRTI] and nonnucleoside reverse transcriptase inhibitor [NNRTI], integrase strand transfer inhibitor [INSTI], or protease inhibitor [PI]), year of ART start, CD4 count at ART start, ART switch during the first 6 months and ADE (either by CD4 count <200 cells/mm^3^, OI, or other clinical event based on the 1993 US Centers for Disease Control and Prevention definition) during the first 6 months since ART initiation were defined as early ADEs (AIDS and Opportunistic Infections, Centers for Disease Control and Prevention, 2021).

CD4 count and viral load at ART start were defined as those measured closest to the date of interest within a period of 90 days before or 7 days after the date of interest (if more than one was recorded, the closest one was included). Patients were excluded from the study if they had an undetectable viral load at or before ART start because this could represent misclassification of their naïve status.

### Statistical analysis

We calculated crude and estimated cumulative incidence of the first LOIs according to a Fine and Gray model, using deaths and LTFU as competing risks events (Fine and Gray, 1999). We also used cumulative incidence curves to describe the incidence of LTFU. Fisher’s exact test or chi-square test was performed for categorical variables and *t*-tests or Wilcoxon rank sum tests for continuous variables, as appropriate.

Risk factor analysis was performed using a multivariable Cox proportional hazards model, and calculation of subdistribution hazard ratios using the previously mentioned competing risks model (Fine and Gray, 1999). In the Cox model, a cause-specific hazards approach was used. Consequently, we censored patients who died or were lost to follow-up. To adjust for probable differences in baseline hazards between sites, the Cox model was stratified by site, and site was included as a covariate in the competing risks model. Age at ART start, year of treatment initiation, and CD4 count at ART start were included in the models, using restricted cubic splines with three knots.

All statistical analyses were done using R software version 4.0.0. The complete analysis code is posted and freely available at https://biostat.app.vumc.org/ArchivedAnalyses.

## Results

**Figure 1.**
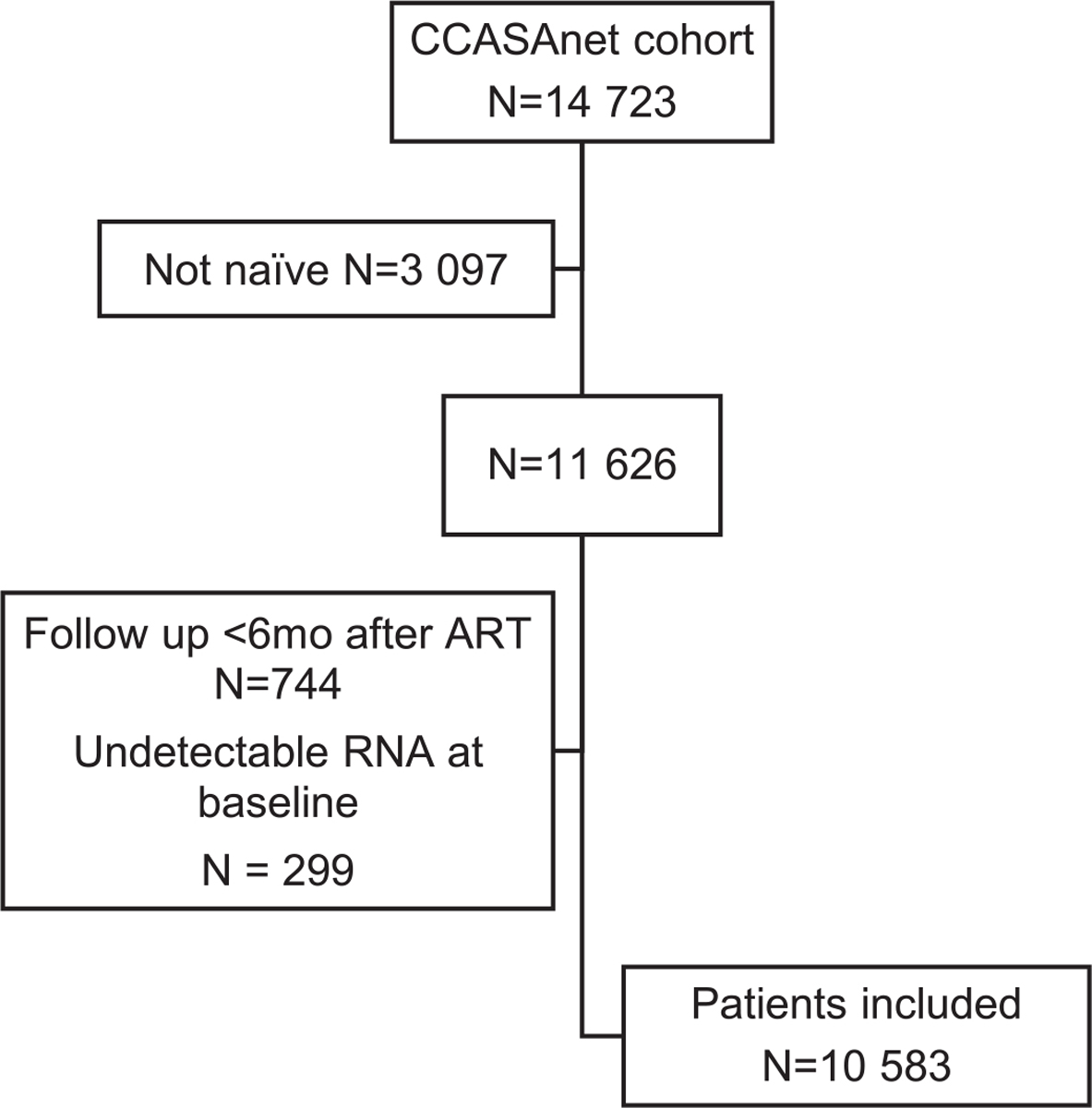
Patient selection flowchart.

Among 14,723 potentially eligible PLWHA enrolled in CCASAnet, 10,583 met the inclusion criteria and were included in the final analysis in this study (Figure 1). Patient distribution was as follows: 484 (4.6%) from Argentina, 2844 (26.9%) from Brazil, 2011 (19%) from Chile, 806 (7.6%) from Honduras, 1031 (9.7%) from Mexico, and 3407 (32.2%) from Peru (Table 1). Median year of ART start was 2010 (IQR 2007–2013, range 2001–2018). Median age was 34 years (IQR 27–42), and 2544 (24%) patients were women. Probable route of HIV infection varied: heterosexual-sex contact in 4748 (44.8%), same-sex contact in 5002 (47.2%), other route in 108 (1%), and unknown in 725 (6.8%). In addition to an NRTI, 8778 (82.9%) patients received an NNRTI, 168 (1.5%) an INSTI, and 1462 (13.8%) a PI as part of their initial combination treatment. Median follow up was 5.4 years (IQR; 2.9–9.1).

**Figure 2.**
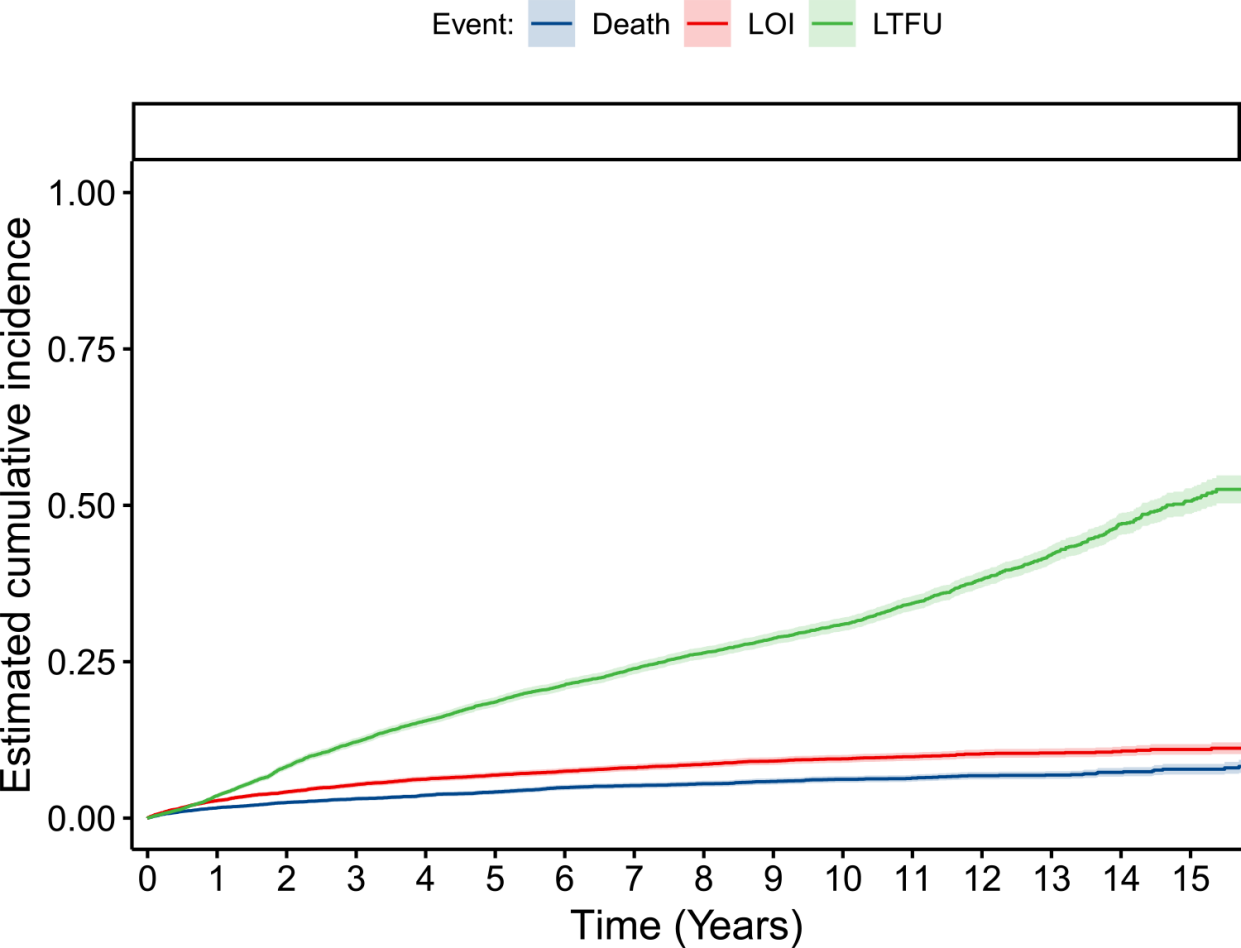
Cumulative incidence estimates for late-onset opportunistic infection, death, and loss to follow-up. A competing risks model as described in the methods section was utilized. LOI = late-onset AIDS-defining opportunistic infection; LTFU = loss to follow-up. Time 0 is 6 months after ART initiation.

Clinical and demographic characteristics, overall and by LOI status, are shown in Table 1. A total of 895 patients (8.4%) had LOIs, 576 (5.4%) died, and 3021 (28.5%) were lost to follow-up. Development of an LOI took a median time of 2.1 years (IQR 0.64–4.5). Crude incidence rates were 1.4 per 100 person-years for LOIs, 0.9 per 100 person-years for death, and 4.6 per 100 person-years for LTFU. Estimated cumulative incidence curves according to the competing risks model are shown in Figure 2. Furthermore, 5, 10, and 15 years after starting ART, the cumulative incidence of LOIs was estimated to be 6.9%, 9.5%, and 11% (respectively); death was estimated at 4.1%, 6.1%, and 7.8%, respectively, whereas LTFU was estimated at 18.5%, 31%, and 50.6%, respectively. A total of 41 patients had two or more simultaneous LOIs as their event. The most common LOIs were tuberculosis (n=359, 38.3%), esophageal candidiasis (n=98, 10.4%), *Pneumocystis jirovecii* (*P. jirovecii*) pneumonia (n=98, 10.4%), toxoplasmosis (n=72, 7.6%), and herpes simplex infection (chronic ulcer, bronchitis, pneumonitis, or esophagitis; n=70, 7.4%). The most common combination was that of *P. jirovecii* pneumonia and esophageal candidiasis in two patients. The proportion of LOIs that were tuberculosis varied according to study center: Peru had 170 (88.1%), Brazil 124 (35.9%), Mexico 14 (30.4%), Chile 28 (21.2%), Argentina 4 (18.2%), and Honduras 16 (10.1%).

A total of 159 (17.8%) patients who developed an LOI had an early ADE, compared with 657 (6.8%) in those who did not. Nine patients had the same LOI as their early ADE. In those who developed an LOI, 47.5% had an undetectable viral load at event or last available date, compared with 84.8% in those who did not have an LOI. CD4 cell count at ART start was lower in the LOI group than in those who did not have an LOI (median 112 cells/mm^3^ vs 200 cells/mm^3^) at the event or last available date (median 190 cells/mm^3^ vs 554 cells/mm^3^, p<0.001). ART suspension (for any reason) or change during the first 6 months of treatment also was more common in patients who developed an LOI (7.5% vs 4%, *P*<0.001).

**Figure 3.**
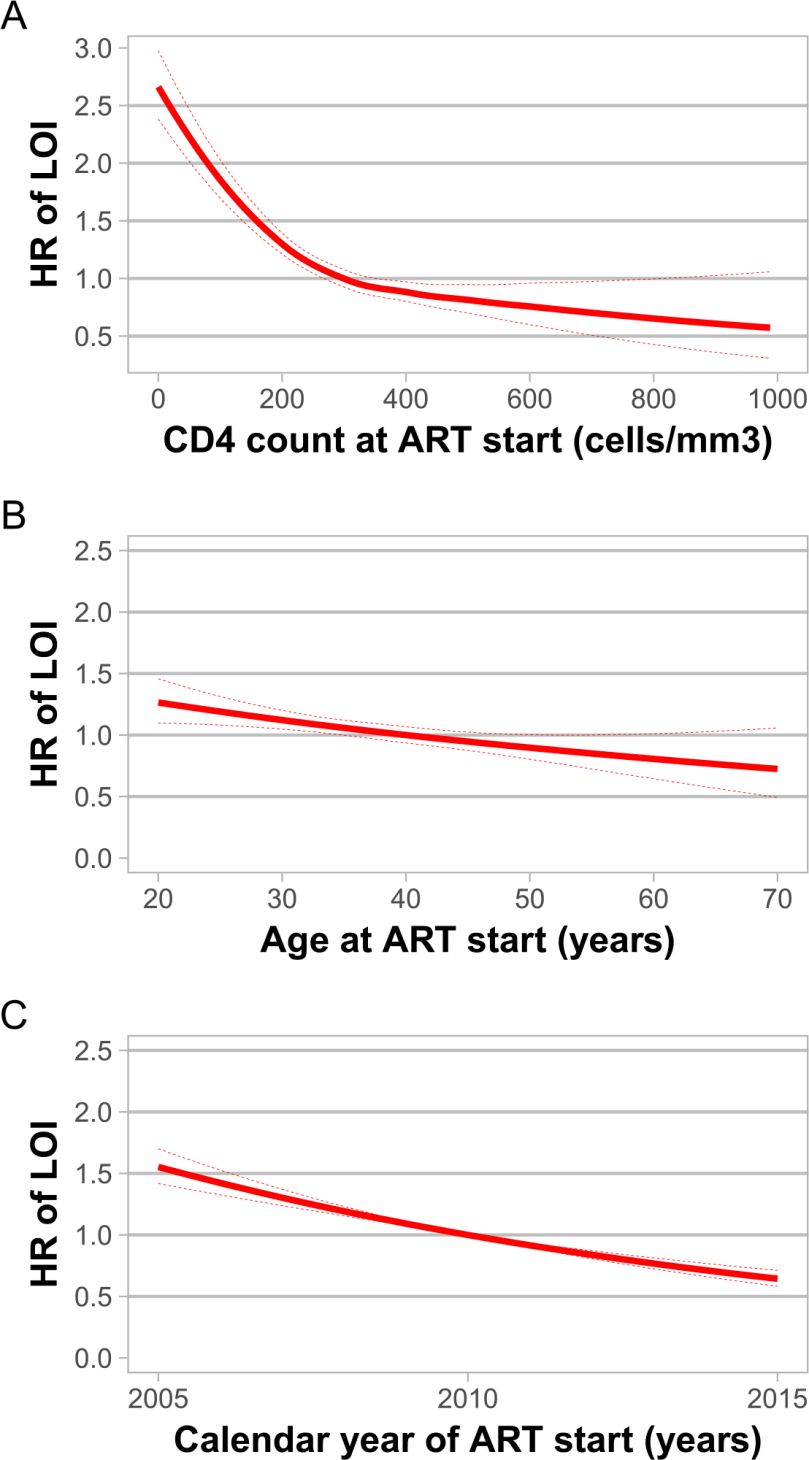
Risk of late-onset opportunistic infection (LOI) according to CD4 cell count (A), age (B), and year (C) of antiretroviral therapy (ART) start. A Cox proportional hazards model was used. HR: hazard ratio. Dashed lines represent 95% confidence limits. CD4 count at ART start was adjusted by age at ART start, and year of ART start; age at ART start was adjusted by year of ART start, and sex; year of ART start was not adjusted by other variables. Cubic splines with three knots (lower quartile-median-upper quartile) were used to adjust CD4 count and age of ART start to better fit the data.

Risk factors included in the multivariate Cox model and hazard ratio subdistribution model are presented in Table 2. In the adjusted model, using a PI-based regimen (subdistribution hazard ratio [sHR]: 1.25; 95%CI 1.02–1.52), having an ADE during the first 6 months of ART (sHR: 2.12, 95%CI: 1.75–2.57), heterosexual mode of transmission (sHR: 1.49; 95%CI: 1.23–1.79), starting ART in early calendar years (sHR: 1.52, 95%CI: 1.5–1.54 for 2005 vs 2010), and ART switch before event (sHR: 1.31, 95%CI: 1.0–1.73) were associated with a higher risk of LOI. Conversely, higher CD4 counts (sHR: 0.7, 95%CI:0.7–0.7 500 cells/mm^3^ vs 300 cells/mm^3^), male sex (sHR: 0.79, 95%CI:0.67–0.93), and older age at ART start (sHR: 0.77, 95%CI:0.76–0.78 60 years vs 40 years) were associated with a lower risk for LOI. The relative risks of an LOI according to CD4 count, age at treatment start, and year of treatment initiation are shown in Figure 3. An increased risk of LOI was consistently observed with lower values of all three variables.

## Discussion

In this analysis of a cohort of more than 10,000 patients from throughout Latin America, crude incidence of LOIs was 1.4 per 100 person-years of follow-up; having an ADE during the first 6 months of ART start, younger age, starting ART in earlier calendar year, ART switch before event, and lower CD4 count at ART start were independently associated with an increased risk for LOIs.

A few studies have analyzed the risk for LOIs. A cohort study in Taiwan of patients newly diagnosed with HIV who started ART reported that approximately 20% of patients developed an AIDS-related opportunistic infection (AOI) after starting ART (Lee *et al*., 2018). Of those who did develop an AOI, approximately 90% of infections occurred during the first 90 days, likely representing an immune-reconstitution or an “unmasking” phenomenon. *P. jirovecii* pneumonia was the most common infection in this study (43%), followed by cytomegalovirus disease (10.4%). Mortality of all AOIs ranged between 14.5% and 20.7% (Lee *et al*., 2018). A Brazilian cohort study explored risk factors for tuberculosis, *P. jirovecii* pneumonia (PJP), esophageal candidiasis, and cerebral toxoplasmosis in PLWHA (Coelho *et al*., 2014). This cohort included those on ART and those not on ART. Incidence rates were highest for tuberculosis (15.3 cases per 1000 person-years) and lowest was for PJP (4.8 per 1000 person-years). Although most cases were reported early during follow-up (first 2 years), diagnoses continued to be made up to 10 years after enrollment (Coelho *et al*., 2014). Importantly, our results parallel theirs, which adds consistency to the common occurrence of LOIs. Common risk factors in their study for all four AOIs were low CD4 nadir and diagnosis of an OI at enrollment, also consistent with our findings.

In our study, for lower CD4 counts at ART initiation, especially <300 cells/mm^3^, a higher risk of LOI was observed that trended down as CD4 count increased. For those in whom CD4 counts were higher than 300 cells/mm^3^, the hazard ratio remained lower than 1. Similarly, age at ART start showed a gradient, with patients aged less than 40 years showing a particularly higher risk for LOI than did older patients. A meta-analysis of aging and adherence to antiretrovirals showed that older adults with HIV have a lower risk for nonadherence than do younger patients (Ghidei *et al*., 2013). This association with age and adherence has been shown previously, although in one meta-analysis of patients in Africa, adherence was comparable in younger and older adults with HIV (Barclay *et al*., 2010; Nachega *et al*., 2009; Soomro *et al*., 2019; Tweya *et al*., 2017). As we observed a lower risk of LOI in all models among older patients, this could probably be attributed to better adherence; however, adherence was not assessed in the present study. In addition, we do not have information on the cause of death, so we cannot make conclusions about causes of death from our data.

Starting ART during earlier calendar years was associated with an increased risk of LOI. Before 2013, most of the guidelines of the region delayed ART initiation until 350 CD4 cell counts, which led to lower CD4 at starting ART, which was also found to be a risk factor associated with LOI. Moreover, the use of restricted ART drugs in the region owing to scarce resources has been previously documented; this implies more complex regimes related to more toxicities and worse adherence that could probably impact on higher risk of LOI (Wolff *et al*., 2016). Moreover, in the region during 2000–2011, we reported a low frequency of laboratory monitoring, causing a delay in virologic failure detection, which could be related to the increase in the risk of LOI (Belaunzarán-Zamudio *et al*., 2015).

Important differences, both at baseline and during follow-up, were present between those who developed an OI and those who did not. Viral suppression was less common among those who developed LOI, being approximately 50% at the time of the OI. This could be owing to loss of adherence to treatment or virologic failure, but because we do not have data on patient adherence or treatment failure (a limitation of this study), we must remain merely speculative. However, it also could be a consequence of the OI. We do not have the data to determine temporality in a strict way. A higher proportion of patients came from Brazil-INI, which could be attributed to better reporting of events.

An association of worse condition at baseline and higher incidence of LOI certainly makes biological sense because worse immune recovery is seen among these patients (Wilson and Sereti, 2013). It is now well established that ART may fail to restore immunologic competence in those who are diagnosed at stage of HIV disease. In a Swiss cohort study, PLWHA who were diagnosed with esophageal candidiasis had impaired immune response years after the event and consistent use of ART (Stuehler *et al*., 2016). We did not measure cytokine or antigen-specific responses, but median CD4 cell count in the patients who developed an LOI was less than 200 (compared with >500 in those who did not develop an LOI), so a similar phenomenon of blunted immunologic response to ART (and thus perhaps increased risk for LOI) seems potentially plausible.

Incidence and type of opportunistic infection differed between ours and other cohorts. Tuberculosis was less common in the previously mentioned Ugandan cohort, affecting only 15.3% of those who developed an OI. Interestingly, they observed an increased risk among those with lower baseline CD4 cell count, as did we, but age was not a significant risk factor in their study (Weissberg *et al*., 2018). This difference could be owing to regional differences in OI incidence or in preferential reporting of one over another given distinct cohort design.

Our study carries several limitations. Data came from a retrospective cohort, with a nonstandardized system to diagnose OIs among countries because of particular local resources, which could be underestimating our findings. We do not have data on latent tuberculosis or on the site of disease to identify extrapulmonary TB, so we cannot make inferences or remarks about it. Thus, the frequency of the precise site of tuberculosis infection may vary between sites, which may have implications for the clinicians performing the follow-up of these patients. Findings may be limited by high LTFU and ascertainment bias. In addition, as mentioned, we did not have specific data on adherence, although we were able to categorize undetectable versus detectable at 6 months after ART start. Nonetheless, our study has several strengths. We included a large representative sample from several countries in Latin America, and most patients had long-term follow-up for more than 5 years.

## Conclusions

In this cohort of people living with HIV in Latin America, LOIs were not uncommon, with nearly 10% in this cohort experiencing an OI more than 6 months after starting antiretroviral therapy. Study limitations include high LTFU and possible ascertainment bias. Nonetheless, LOIs continue to be diagnosed years after starting ART. Virologic failure and blunted immune response are likely causes that deserve further study. ART initiation alone may not be sufficient for “return to health,” and ongoing efforts must be made toward adherence and retention in care.

## Figures and Tables

**Table 1:** Patient clinical and sociodemographic characteristics

Variable	Did not develop a LOI (% or IQR)	Developed a LOI(% or IQR)	*P*-value

Total count (n = 10 583)	9688 (100%)	895 (100%)	-
Median time of follow up/to event (years)	3.5 (1.7–6.9)	2.1 (0.7–4.5)	< 0.001
Age	34 (27–42)	33 (27–41)	0.521
Female	2276 (23.5%)	268 (29.9%)	< 0.001
**Site**	-	-	< 0.001
Argentina	462 (4.8%)	22 (2.5%)	-
Brazil	2499 (25.8%)	345 (38.5%)	-
Chile	1879 (19.4%)	132 (14.7%)	-
Honduras	649 (6.7%)	157 (17.5%)	-
Mexico	985 (10.2%)	46 (5.1%)	-
Peru	3214 (33.2%)	193 (21.6%)	-
**Mode of transmission**	-	-	< 0.001
MSM	4689 (48.4%)	313 (35%)	-
Heterosexual	4276 (44.1%)	472 (52.7%)	-
Other	94 (1%)	14 (1.6%)	-
Unknown	629 (6.5%)	96 (10.7%)	-
**ART includes:**	-	-	< 0.001
Non nucleoside retrotranscriptase inhibitor	8051 (83.1%)	730 (81.6%)	-
Integrase inhibitor	159 (1.6%)	9 (1%)	-
Protease inhibitor	1324 (13.7%)	138 (15.4%)	-
**Year of ART start**	2011 (2007–2013)	2007 (2004–2011)	< 0.001
Early ADE (< 6 months)	657 (6.8%)	159 (17.8%)	< 0.001
LTFU	3021 (31.2%)	-	-
Deaths	580 (6%)	-	-
**Undetectable viral load (percentage <200 copies/mm^3^)**	-	-	-
At 6 months of ART (n = 1188)	991 (10.2%)	49 (5.5%)	< 0.001
At event or last available (n = 10 534)	8218 (84.8%)	425 (47.5%)	< 0.001
**CD4 cell count (cells/mm^3^)**	-	-	-
At ART start (n =9250)	200 (79–326)	112 (37–235)	< 0.001
At event or last available (n = 10575)	554 (365–759)	190 (69–352)	< 0.001
Suspended/changed ART prior to event	806 (8.3%)	97 (10.7%)	0.015
Suspended/changed ART during the first 6 months of treatment	384 (4%)	67 (7.5%)	< 0.001

ART = antiretroviral therapy; IQR = interquartile range; LOI = late-onset opportunistic infection; MSM = men who have sex with men; n = number of patients for whom the value was available.

Median comparisons were made with median test; Wilcoxon’s rank sum was used for continuous variables and chi squared test for categorical variables. Follow-up durations were compared with median test.

**Table 2:** Adjusted risk of late-onset AIDS-defining opportunistic infections

Variable	Adjusted HR(95% Cl)	Subdistribution HR(95% Cl)

**Male sex**	1.06 (0.89–1.27)	0.79 (0.67–0.93)
**ART includes:**	-	-
**NNRTI**	Reference	Reference
**INSTI**	1.3 (1.06–1.6)	1.25 (1.02–1.52)
**PI**	0.91 (0.59–1.4)	0.96 (0.62–1.48)
**Age at ART start:**	-	-
20	1.53 (1.27–1.84)	1.29 (1.28–1.3)
30	1.19 (1.13–1.26)	1.14 (1.13–1.15)
40	Reference	Reference
50	0.9 (0.8–1.01)	0.88 (0.87–0.89)
60	0.82 (0.64–1.06)	0.77 (0.76–0.78)
**ADE during first 6 months of ART**	1.71 (1.41–2.09)	2.12 (1.75–2.57)
**Mode of transmission:**	-	-
**MSM**	Reference	Reference
**Heterosexual contact**	1.31 (1.09–1.58)	1.49 (1.23–1.79)
**Unknown**	1.82 (1.03–3.23)	2.09 (1.16–3.75)
**Other**	1.28 (0.96–1.72)	1.65 (1.27–2.15)
**Year of ART start:**	-	-
2005	1.34 (1.21–1.5)	1.52 (1.5–1.54)
2010	Reference	Reference
2015	0.74 (0.67–0.83)	0.66 (0.64–0.68)
**CD4 count at ART start**	-	-
100 cells/mm^3^	1.67 (1.57–1.79)	1.43 (1.43–1.43)
200 cells/mm^3^	1.23 (1.13–1.33)	1.2 (1.2–1.2)
300 cells/mm^3^	Reference	Reference
400 cells/mm^3^	0.88 (0.81–0.97)	0.84 (0.84–0.84)
500 cells/mm^3^	0.81 (0.69–0.94)	0.7 (0.7–0.7)
**ART switch prior to event**	1.31 (0.99–1.72)	1.31 (1–1.73)

ART: antiretroviral therapy; CI: confidence interval; HR: hazard ratio; MSM: men who have sex with men; NNRTI: nonnucleoside reverse transcriptase inhibitor; PI: protease inhibitor. Adjusted HR refers to a multivariate Cox proportional hazards model in which all the variables in the table were included. Subdistribution hazard ratios refer to those obtained with a competing risk model as described by Fine and Gray, in which all variables in the table were included. The Cox model was stratified by site, and the competing risk model was adjusted by site.
